# Maternal exposure to ambient fine particulate matter and risk of premature rupture of membranes in Wuhan, Central China: a cohort study

**DOI:** 10.1186/s12940-019-0534-y

**Published:** 2019-11-14

**Authors:** Kun Wang, Yu Tian, Huabo Zheng, Shengshuai Shan, Xiaofang Zhao, Chengyun Liu

**Affiliations:** 1 Department of Geriatrics, Union Hospital, Tongji Medical College, Huazhong University of Science and Technology, Wuhan, 430022 China; 2 Organisation for Economic Co-operation and Development, 92100 Boulogne-Billancourt, France; 3 The First People’s Hospital of Jiangxia District, Wuhan City & Union Jiangnan Hospital, HUST, Wuhan, 430200 China

**Keywords:** Cohort study, Maternal exposure, PM_2.5_ (airborne particulate matter with an aerodynamic diameter of 0.25 μm or less), PROM (premature rupture of membranes), PPROM (preterm premature rupture of membranes)

## Abstract

**Background:**

The associations between maternal exposure to ambient PM_2.5_ during pregnancy and the risk of premature rupture of membranes (PROM) and preterm premature rupture of membranes (PPROM) are controversial. And no relevant study has been conducted in Asia. This study aimed to determine the association between maternal exposure to ambient PM_2.5_ during pregnancy and the risk of (P)PROM.

**Methods:**

A cohort study including all singleton births in a hospital located in Central China from January 2015 through December 2017 was conducted. Multivariable logistic regression models, stratified analysis, generalized additive model, and two-piece-wise linear regression were conducted to evaluate how exposure to ambient PM_2.5_ during pregnancy is associated with the risks of PROM and PPROM.

**Results:**

A total of 4364 participants were included in the final analysis, where 11.71 and 2.34% of births were complicated by PROM and PPROM, respectively. The level of PM_2.5_ exhibited a degree of seasonal variation, and its median concentrations were 63.7, 59.3, 55.8, and 61.8 μg/m^3^ for the first trimester, second trimester, third trimester, and the whole duration of pregnancy, respectively. After adjustment for potential confounders, PROM was positively associated with PM_2.5_ exposure (per 10 μg/m^3^) [Odds Ratio (OR) = 1.14, 95% Confidence Interval (CI), 1.02–1.26 for the first trimester; OR = 1.09, 95% CI, 1.00–1.18 for the second trimester; OR = 1.13, 95% CI, 1.03–1.24 for the third trimester; OR = 1.35, 95% CI, 1.12–1.63 for the whole pregnancy]. PPROM had positive relationship with PM_2.5_ exposure (per 10 μg/m^3^) (OR = 1.17, 95% CI, 0.94–1.45 for first trimester; OR = 1.11, 95% CI, 0.92–1.33 for second trimester; OR = 1.19, 95% CI, 0.99–1.44 for third trimester; OR = 1.53, 95% CI, 1.03–2.27 for the whole pregnancy) Positive trends between the acute exposure window (mean concentration of PM_2.5_ in the last week and day of pregnancy) and risks of PROM and PPROM were also observed.

**Conclusions:**

Exposure to ambient PM_2.5_ during pregnancy was associated with the risk of PROM and PPROM.

## Introduction

The membranes surrounding the amniotic cavity normally rupture at the beginning of labor or during labor. Premature rupture of membranes (PROM) is defined as rupture of membranes (ROM) that occurs more than 1 h before the onset of labor [1]. If rupture occurs before 37 weeks, it is considered preterm PROM (PPROM). PROM, especially PPROM, has been linked with a number of adverse outcomes, including preterm birth, chorioamnionitis, endomyometritis, pelvic abscess, bacteremia, postpartum hemorrhage [2, 3], umbilical cord prolapses, umbilical cord compression, retained placenta, fetal distress [4, 5], and early onset neonatal infection [6]. These outcomes are often associated with increased maternal complications, neonatal mortality [7–9], and even adverse long-term outcomes [10].

Despite the high incidence and harmful consequences, the etiology of (P)PROM remains unclear to some degree. Recently, Pereira et al. found that exposure to airborne particulate matter with an aerodynamic diameter of 0.25 μm or less (PM_2.5_) during the second trimester of pregnancy had an effect on PROM risk in Western Australia [11]. But Dadvand et al. demonstrated that the increased risk of PPROM was associated with PM_2.5_ absorbance but not with PM_2.5_ exposure itself [12]. Consequently, the relationship between PM_2.5_ exposure and (P)PROM is controversial. Meanwhile, to our knowledge, these previous similar studies were all conducted in Caucasian whose environmental status is higher than the national average. No available epidemiological study has discussed the link between PM_2.5_ and the risks of (P)PROM in China, a country with a considerably high level of PM_2.5_.

In the present study, we aimed to investigate the association if any between exposure to ambient PM_2.5_ during pregnancy and the incidence of PROM and PPROM in Wuhan, Central China.

## Patients and methods

### Study population

**Fig. 1:**
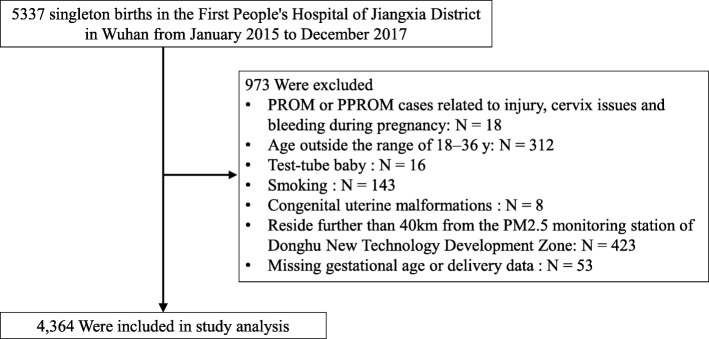
Flow chart of the cohort study

We conducted a population-based study using hospital database in the First People’s Hospital of Jiangxia District in Wuhan, which is a major hospital in the Jiangxia District. We constructed a study cohort that included all singleton births from January 2015 to December 2017. A flow chart (Fig. 1) of the exclusion criteria was provided. Exclusion criteria for participants were as follows: (a) PROM or PPROM cases related to injury, cervix issues, and bleeding during pregnancy; (b) individuals with ages outside the range of 18–35 years old; (c) test tube babies; (d) former or current smokers; (e) congenital uterine malformations; (f) residence in areas further than 40 km from the PM_2.5_ monitoring station of Donghu New Technology Development Zone; and (g) missing gestational age or delivery data. The final analyses included 4364 singleton live births.

The study protocol was approved by the Medical Ethics Committee of the First People’s Hospital of Jiangxia District and complied with the Declaration of Helsinki. We verbally informed the participants that the data will be used anonymously for a medical study. No informed consent was required, because the study was observational, and the data were anonymized.

### Measurement of variables

In China, pregnant women are advised to start systematic antenatal examination and hospital record at the end of the first trimester (i.e., week 12). The hospital records contain a wide range of clinical data on pregnancy and delivery, together with demographic characteristics, underlying diseases, and previous medical histories. ROM diagnosis was performed using the following: pooling test, which is the collection of fluid in the vaginal fornix or fluid leaking from the cervical opening (during coughing or valsalva maneuver); and Nitrazine test, which uses fluid collected from the posterior fornix of the vagina with an alkaline pH. The first and second trimesters were defined as weeks 1–12 and 13–27, respectively. The third trimester was defined as commencing at week 28 and ending at week 40 or at birth, whichever was earlier.

### Exposure assignment

Daily (24-h average) PM_2.5_ measurements were obtained from the Ministry of Ecological Environment of the People’s Republic of China (Donghu New Technology Development Zone site, 114.3894 °E, 30.4822 °N). This site is the nearest monitoring station in the First People’s Hospital of Jiangxia District (approximately 13 km) and the only monitor operational for PM_2.5_ measurements throughout the study period. We excluded puerperae residing outside 40 km of the vicinity of the site. This choice of threshold distance is same with that used in the study conducted by Pereira in 2014 [11], and is shorter than that used for sensitivity analysis in the study of Di in 2017 [13]. For each subject, we computed the average concentration of PM_2.5_ for the whole pregnancy, each trimester, the last week, and last day of pregnancy. Temperature exposures were generated by using the same procedure described for PM2.5 exposure.

### Statistical analyses

**Fig. 2:**
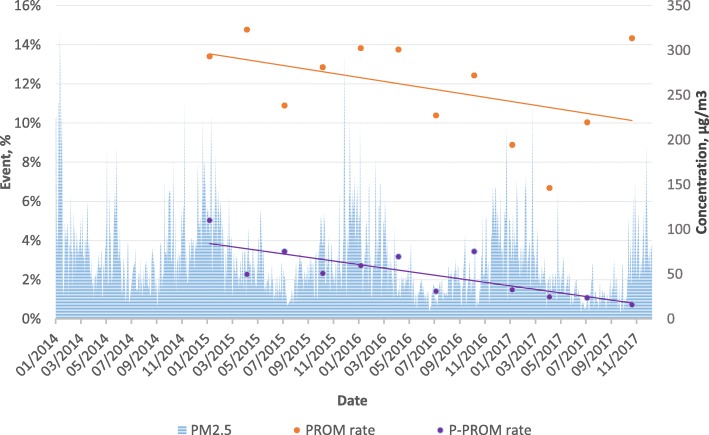
Temporal trend of PM_2.5_ level and quarterly incidence rates of PROM and PPROM from January 2014 to December 2017

The quarterly incidence rates of PROM and PPROM were calculated and compared with the temporal trend of PM_2.5_ (Fig. 2). The summary statistics of the characteristics of all patients were expressed as frequencies (proportions) for categorical variables and as means ± SD or median (interquartile range) for continuous variables (Table 1). We also conducted a univariate analysis to evaluate the association between the characteristics and (P)PROM (Additional file 1: Table S1).

**Table 1:** Characteristics of participants

Characteristics	All Births (*N* = 4364)	PROM (*N* = 511)	PPROM (*N* = 102)
Mode of delivery			
Vaginal	3267 (74.9%)	396 (77.5%)	88 (86.3%)
Cesarean	1097 (25.1%)	115 (22.5%)	14 (13.7%)
Year of birth			
2015	1674 (38.3%)	210 (41.1%)	49 (48.0%)
2016	1540 (35.3%)	185 (36.2%)	39 (38.2%)
2017	1150 (26.4%)	116 (22.7%)	14 (13.7%)
Season of conception^a^			
Spring	976 (22.4%)	115 (22.5%)	22 (21.6%)
Summer	1001 (22.9%)	115 (22.5%)	22 (21.6%)
Autumn	1158 (26.5%)	122 (23.9%)	26 (25.5%)
Winter	1229 (28.2%)	159 (31.1%)	32 (31.4%)
Maternal age	26.7 ± 3.6	26.6 ± 3.5	26.3 ± 3.6
Maternal age group			
18–19	66 (1.5%)	7 (1.4%)	4 (3.9%)
20–24	1212 (27.8%)	135 (26.4%)	25 (24.5%)
25–29	2129 (48.8%)	272 (53.2%)	56 (54.9%)
30–35	957 (21.9%)	97 (19.0%)	17 (16.7%)
Parity			
0	3751 (85.9%)	467 (91.4%)	96 (94.1%)
≥ 1	613 (14.1%)	44 (8.6%)	6 (5.9%)
Maternal anemia			
No	4045 (92.7%)	474 (92.8%)	95 (93.1%)
Yes	319 (7.31%)	37 (7.2%)	7 (6.9%)
Maternal preeclampsia			
No	4308 (98.7%)	490 (95.9%)	90 (88.2%)
Yes	56 (1.3%)	21 (4.1%)	12 (11.8%)
Maternal gestational diabetes			
No	4175 (95.7%)	502 (98.2%)	101 (99.0%)
Yes	189 (4.3%)	9 (1.8%)	1 (1.0%)
History of obstetrical-gynecological pathology^b^			
No	4173 (95.6%)	493 (96.5%)	100 (98.0%)
Yes	191 (4.4%)	18 (3.5%)	2 (2.0%)
Mean temperature across the whole pregnancy (°C)	18.0 ± 2.3	18.0 ± 2.3	18.0 ± 2.4
Mean concentration of PM2.5 in the 1st trimester (μg/m^3^)	63.7 (47.6–85.6)	64.4 (50.9–84.5)	65.3 (53.2–86.9)
Mean concentration of PM2.5 in the 2nd trimester (μg/m^3^)	59.3 (41.7–81.4)	59.2 (41.8–81.0)	59.5 (42.0–82.3)
Mean concentration of PM2.5 in the 3rd trimester (μg/m^3^)	55.8 (39. 9–73.8)	55.0 (40.1–79.0)	59.2 (43.7–79.0)
Mean concentration of PM2.5 across the whole pregnancy (μg/m^3^)	61.8 (55.4–67.8)	62.7 (55.8–68.0)	65.9 (56.2–70.5)

Data are expressed in *N* (%) or mean ± SD or Median (interquartile range)

*PROM* Premature rupture of membranes, *PPROM* Preterm premature rupture of membranes

^a^Spring: March to May; Summer: June to August; Autumn: September to November; Winter: December to February

^b^Including chorioamnionitis, uterine myoma, adnexal cyst, pelvic infection, pelvic pathologic adhesion, pelvicellulitis, cervicitis, vaginal bleeding during pregnancy, and/or colpomycosis

**Fig. 3:**
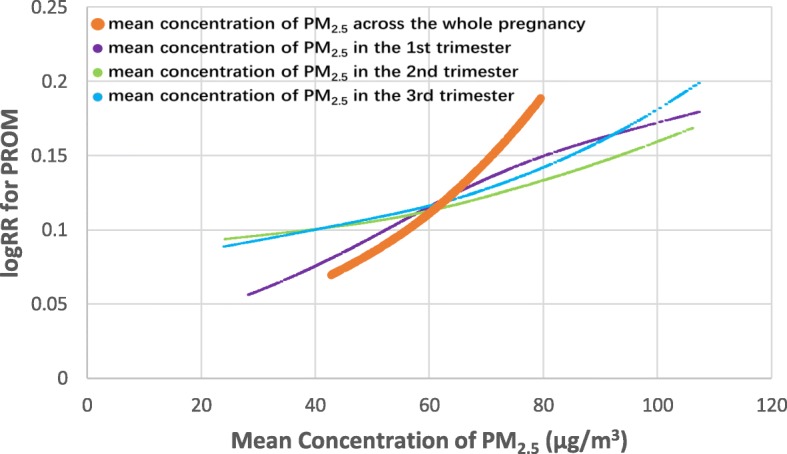
Smooth curves between the mean concentration of PM_2.5_ for each trimester and for the whole pregnancy and PROM*. *The orange line represents the association between the mean concentration of PM_2.5_ during the whole pregnancy and PROM, adjusted for year of birth, season of conception, maternal age, parity, maternal anemia, preeclampsia, gestational diabetes, and history of obstetrical-gynecological pathology (Model I); The purple line represents the association between the mean concentration of PM_2.5_ during the first trimester and PROM, adjusted for Model I plus mean concentration of PM_2.5_ in the second and third trimesters; The green line represents the association between the mean concentration of PM_2.5_ during the second trimester and PROM, adjusted for Model I plus mean concentration of PM_2.5_ in the first and third trimesters; The blue line represents the association between the mean concentration of PM_2.5_ during the third trimester and PROM, adjusted for Model I plus mean concentration of PM_2.5_ in the first and second trimesters

**Fig. 4:**
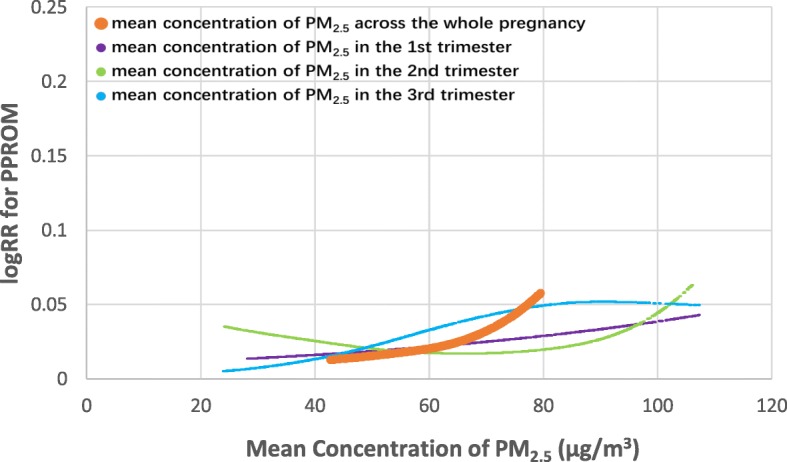
Smooth curves between the mean concentration of PM_2.5_ for each trimester and the whole pregnancy and PPROM*. *The orange line represents the association between the mean concentration of PM_2.5_ during the whole pregnancy and PPROM, adjusted for year of birth, season of conception, maternal age, parity, maternal anemia, preeclampsia, gestational diabetes, and history of obstetrical-gynecological pathology (Model I); The purple line represents the association between the mean concentration of PM_2.5_ during the first trimester and PPROM, adjusted for Model I plus mean concentration of PM_2.5_ in second and third trimesters; The green line represents the association between the mean concentration of PM_2.5_ during the second trimester and PPROM, adjusted for Model I plus mean concentration of PM_2.5_ in the first and third trimesters; The blue line represents the association between the mean concentration of PM_2.5_ during the third trimester and PPROM, adjusted for Model I plus mean concentration of PM_2.5_ in the first and second trimester

Logistic regression models were used to evaluate the relationships of the concentration of PM_2.5_ (per 10 μg/m^3^) in each trimester and in the whole pregnancy (as continuous variables) to the risks of PROM and PPROM with and without adjustment for confounding variables (year of birth, season of conception, maternal age, parity, maternal anemia, preeclampsia, gestational diabetes, history of obstetrical-gynecological pathology, and/or mean concentration of PM_2.5_ in the other two trimesters). We calculated the hazard odd ratios (ORs) and 95% confidence intervals (CIs) (Table 2). We then applied generalized additive models to estimate the above-mentioned relationships with adjustment for potential confounders (Figs. 3 and 4; Additional file 1: Figures S1–S6).

**Table 2:** ORs (95%CI) for PROM and PPROM per 10 μg/m^3^ Increase in PM2.5 in each trimester and across the whole pregnancy (*N* = 4364)

Crude		Model I^a^	Model II^b^
PROM			
1st trimester	1.02 (0.97, 1.06)	1.00 (0.95, 1.05)	1.14 (1.02, 1.26) ^c^
2nd trimester	1.00 (0.96, 1.05)	1.09 (1.00, 1.18)	1.09 (1.00, 1.18) ^d^
3rd trimester	1.03 (0.99, 1.08)	1.03 (0.98, 1.08)	1.13 (1.03, 1.24) ^e^
Whole pregnancy	1.09 (0.99, 1.20)	1.35 (1.12, 1.63)	–
PPROM			
1st trimester	1.04 (0.95, 1.13)	0.97 (0.87, 1.09)	1.17 (0.94, 1.45) ^c^
2nd trimester	1.05 (0.96, 1.14)	1.12 (0.93, 1.35)	1.11 (0.92, 1.33) ^d^
3rd trimester	1.07 (0.98, 1.18)	1.07 (0.97, 1.18)	1.19 (0.99, 1.44) ^e^
Whole pregnancy	1.31 (1.06, 1.62)	1.53 (1.03, 2.27)	–

*OR* Odds Ratio, *CI* Confidence Interval

*PROM* Premature rupture of membranes, *PPROM* Preterm premature rupture of membranes

^a^Model I adjusted for: year of birth, season of conception, maternal age, parity, maternal anemia, preeclampsia, gestational diabetes, and history of obstetrical-gynecological pathology

^b^Model II adjusted for Model I plus mean concentration of PM2.5 in the other two trimesters

^c^Model I plus mean concentration of PM2.5 in the 2nd and 3rd trimesters

^d^Model I plus mean concentration of PM2.5 in the 1st and 3rd trimesters

^e^Model I plus mean concentration of PM2.5 in the 1st and 2nd trimesters

As part of a sensitivity analysis, we did stratified analysis. We conducted logistic regression models to evaluate the relationships of the concentration of PM_2.5_ (per 10 μg/m^3^) in the whole pregnancy to the risks of (P)PROM with adjustment for above-mentioned confounding variables in subgroups of age, parity, mode of delivery, season of conception, maternal anemia, preeclampsia, gestational diabetes and history of obstetrical-gynecological pathology (Additional file 1: Table S2).

The associations were further investigated using a two-piece-wise linear model. The turning point of PM_2.5_, where the relationship between incidence of PROM or PPROM and PM_2.5_ started to change and became eminent was determined using trial method, which was to move the trial turning point along the pre-defined interval and selected the one that provides the maximum model likelihood. For convenient clinical use, we designated the nearest half or whole number as the turning point (Table 3).

**Table 3:** Threshold Effect for PROM and PPROM per 10 μg/m^3^ Increase in PM2.5 across the whole pregnancy (*N* = 4364)^a^

	ORs (95% CI)
PROM	
PM2.5 < 46 (μg/m^3^)	0.18 (0.03, 1.34)
PM2.5 ≥ 46 (μg/m^3^)	1.43 (1.17, 1.73)
PPROM	
PM2.5 < 63 (μg/m^3^)	1.15 (0.71, 1.85)
PM2.5 ≥ 63 (μg/m^3^)	2.42 (1.31, 4.46)

*OR* Odds Ratio, *CI* Confidence Interval

*PROM* Premature rupture of membranes, *PPROM* Preterm premature rupture of membranes

^a^Adjusted for: year of birth, season of conception, maternal age, parity, maternal anemia, preeclampsia, gestational diabetes, and history of obstetrical-gynecological pathology (Model I)

We also investigated the association between acute exposure windows (mean concentration of PM_2.5_ in the last week and day of pregnancy) and ROM. The results are presented in Table 4 and Additional file 1: Figures S7–S10. PM_2.5_ effect estimates were calculated per 10 μg/m^3^ increment and as quartiles.

**Table 4:** Adjusted ORs (95% CI) for PROM and PPROM associated with 10 μg/m^3^ Increase in PM2.5 and with quartiles of PM2.5 (relative to the lowest quartile) in the last week and last day of pregnancy (*N* = 4364)^a^

	PROM	PPROM
Last week of pregnancy		
PM2.5 (10 μg/m^3^)	1.01 (0.97, 1.04)	1.04 (0.96, 1.12)
< 37.5	1.0	1.0
37.5 to < 75	0.79 (0.60, 1.05)	1.01 (0.55, 1.85)
75 to < 112.5	0.84 (0.63, 1.11)	0.83 (0.43, 1.59)
≥112.5	1.00 (0.74, 1.34)	1.36 (0.71, 2.61)
Last day of pregnancy		
PM2.5 (10 μg/m^3^)	1.02 (1.00, 1.05)	1.04 (0.99, 1.10)
< 59	1.0	1.0
59 to < 118	0.99 (0.75, 1.30)	0.95 (0.52, 1.74)
118 to < 177	0.90 (0.68, 1.20)	0.96 (0.51, 1.78)
≥177	1.28 (0.96, 1.69)	1.44 (0.78, 2.66)

*OR* Odds Ratio, *CI* Confidence Interval

*PROM* Premature rupture of membranes, *PPROM* Preterm premature rupture of membranes

^a^Adjusted for: year of birth, season of conception, maternal age, parity, maternal anemia, preeclampsia, gestational diabetes, and history of obstetrical-gynecological pathology

The temporal trend of temperature and the associations betwS7een temperature and PM_2.5_ and (P)PROM were also presented in Additional file 1: Figures S11–S13 and Additional file 1: Table S3. Variance Inflation Factor test was conducted to evaluate the multicollinearity between the potential risk factors and independent variable in Additional file 1: Table S4. Temperature exposure was included in adjusted models to assess sensitivity of the observed odds ratios (Additional file 1: Tables S5-S7).

Data were analyzed using the statistical packages R (R Foundation; http://www.r-project.org; version 3.4.3) and EmpowerStats (www.empowerstats.com; X&Y Solutions Inc.).

## Results

During the study period, 11.7% (511) and 2.3% (102) of births were complicated by PROM and PPROM, respectively. The median concentrations of PM_2.5_ were 63.7, 59.3, 55.8, and 61.8 μg/m^3^ for the first trimester, second trimester, third trimester, and the whole duration of pregnancy, respectively (Table 1). Degrees of seasonal variation were observed in the level of PM_2.5_ and in the incidence rates of PROM and PPROM. The latter exhibited decreasing tendencies that were not observed in the air quality data (Fig. 2).

The mean age of the puerperae was 26.7 years. PROM and PPROM case births exhibited higher proportion of vaginal delivery, conception in winter, firstborn and maternal preeclampsia compared with births of women without (P) PROM (Table 1).

Table 2 shows that when adjusted for year of birth, season of conception, maternal age, parity, maternal anemia, preeclampsia, gestational diabetes, and history of obstetrical-gynecological pathology (Model I), the mean PM_2.5_ values (per 10 μg/m^3^) during the whole pregnancy were positively associated with PROM risk (OR = 1.35, 95% CI, 1.12–1.63) and PPROM (OR = 1.53, 95% CI, 1.03–2.27). But the relationships between PM_2.5_ in each trimester and the risks of (P)PROM were not significant. Given that the level of PM_2.5_ exhibited a degree of seasonal variation, the effects during the two trimesters with relatively low PM_2.5_ level were still considerable and were not overshadowed by the trimester with the highest PM_2.5_ level. Therefore, we adjusted mean PM_2.5_ in the other two trimesters to estimate the independent relationships (Model II). In Model II, mean PM_2.5_ values (per 10 μg/m^3^) in every trimester were positively associated with PROM risk (OR = 1.14, 95% CI, 1.02–1.26 for first trimester; OR = 1.09, 95% CI, 1.00–1.18 for second trimester; OR = 1.13, 95% CI, 1.03–1.24 for third trimester). The relationships between mean PM_2.5_ values (per 10 μg/m^3^) in every trimester and PPROM still had positive trends (OR = 1.17, 95% CI, 0.94–1.45 for first trimester; OR = 1.11, 95% CI, 0.92–1.33 for second trimester; OR = 1.19, 95% CI, 0.99–1.44 for third trimester).

In the sensitivity analysis, we found the independent positive relationships between the concentration of PM_2.5_ in the whole pregnancy and (P)PROM were robust and consistent in different subgroups of age, parity, mode of delivery, season of conception, maternal anemia, preeclampsia, gestational diabetes and history of obstetrical-gynecological pathology (Additional file 1: Table S2).

Figures 3 and 4 show the nonlinear relationships of the concentration of PM_2.5_ in each trimester and in the whole pregnancy to PROM and PPROM by curve fitting (separate curves of different adjusted models are presented in Additional file 1: Figures S1–S6).

By two-piece-wise linear regression, in the present study we found the turning points of 46 and 63 μg/m^3^ for PROM and PPROM, respectively. For PROM, the adjusted OR was 1.43 (95% CI, 1.17–1.73) when PM_2.5_ ≥ 46 μg/m^3^ and 0.18 (95% CI, 0.03–1.34) when PM_2.5_ < 46 μg/m^3^. For PPROM, the adjusted OR values were 2.42 (95% CI, 1.31–4.46) and 1.15 (95% CI, 0.71–1.85) when PM_2.5_ ≥ 63 μg/m^3^ and PM_2.5_ < 63 μg/m^3^, respectively (Table 3).

Positive trends between the acute exposure window (mean concentration of PM_2.5_ in the last week and day of pregnancy) and the risks of PROM and PPROM were observed. As for PROM, OR = 1.01 (95% CI, 0.97–1.04) for last week of pregnancy and OR = 1.02 (95% CI, 1.00–1.05) for last day of pregnancy (per 10 μg/m3). As for PPROM, OR = 1.04 (95% CI, 0.96–1.12) for last week of pregnancy and OR = 1.04 (95% CI, 0.99–1.10) for Last day of pregnancy (per 10 μg/m3) (Table 4). The nonlinear relationships between acute exposure to PM_2.5_ and the risks of PROM and PPROM are presented in Additional file 1: Figures S7–S10.

Additional file 1: Figure S11 showed a degree of seasonal variation level of the temperature. Additional file 1: Table S3 showed that there is no significant relationship between temperature and (P)PROM. We found that there were severe multicollinearities (Variance Inflation Factor > 5) between temperature and PM2.5 during pregnancy (Additional file 1: Table S4). Sensitivity analysis to temperature adjustment showed that the ORs and turning points did not change considerably after adjustment for temperature exposures, while the 95% CIs got a little wider (Additional file 1: Tables S5-S7).

## Discussion

In this population-based cohort study, our multilevel analysis provided preliminary evidence that exposure to PM_2.5_ during pregnancy was significantly associated with increased risks of PROM and PPROM in Wuhan, Central China. To our knowledge, this is the first study of the relationship between PM_2.5_ and (P)PROM in Asia and areas with relatively high air pollution.

Previous studies have also investigated the associations between PM_2.5_ and the risks of PROM and PPROM, but the results were controversial. In the study conducted in Barcelona between 2002 and 2013 (median PM_2.5_ was 19.8 μg/m^3^), Dadvand et al. demonstrated that the increased risk of PPROM was associated with PM_2.5_ absorbance but not with PM_2.5_ exposure itself [12]. In the study conducted in Western Australia from 1997 to 2007(median PM_2.5_ was 8.55 μg/m^3^), Pereira et al. found that PROM was only significantly associated with PM_2.5_ exposure in the second trimester but not in the first or third trimester or the whole pregnancy [11]. In a retrospective cohort study in America (median PM_2.5_ was 11.9 μg/m^3^), Maeve et al. identified associations during acute windows of elevated exposure to PM_2.5_ in the last 3 h before delivery, but no evidence exists on the association between whole-pregnancy exposure to PM_2.5_ and PROM or PPROM [14].

In the present study, given the more severe air pollution in China and the use of different statistical methods, we found significant association between PM_2.5_ exposure and the risks of PROM and PPROM. In our study, the values of PM_2.5_ were relatively high, and had almost no overlap with the previous studies (the median concentration of 61.8 μg/m^3^ vs 19.8 μg/m^3^, 8.55 μg/m^3^and 11.9 μg/m^3^). An analysis of PM_2.5_ levels in 22 countries by the World Health Organization found an association with preterm birth only in China—the country with the highest levels of PM_2.5_ [15], which may be similar to our situation. As for statistical analyses, compared with previous studies where the date was treated as a whole, we analyzed the data segmentally according to the turning points to obtain more accurate results. At the same time, adjusting the mean PM_2.5_ in the other two trimesters (Model II) can counteract the seasonal fluctuations in the PM_2.5_, which may have a huge impact on results. However, previous studies didn’t make such an attempt. These may partly explain the negative results of previous similar studies.

The association between PM_2.5_ and the risks of PROM and PPROM could be partially explained by oxidative stress mechanism. As a leading air pollutant, PM_2.5_ can lodge deep inside our lungs and enter the blood stream, causing respiratory, cardiovascular, cerebrovascular [16], and kidney diseases [17], as well as adverse pregnancy outcomes. A newly published study showed independent associations between exposure to PM2.5 and daily all-cause, cardiovascular, and respiratory mortality in more than 600 cities across the globe [18]. Studies have shown that maternal exposure to PM_2.5_ during the prenatal period was associated with abortion, preterm death and birth, low birth weight, intrauterine growth defects, placental DNA hypomethylation and mtDNA methylation [19–24]. Multiple studies indicated that PM_2.5_ can induce or increase oxidative stress and oxidative DNA damages in the human body [25, 26]. Growing evidence show that oxidative stress plays a role in the pathogenesis of reproduction [27]. Specifically, exposure to PM_2.5_ could induce the production of reactive oxygen species, which damage DNA [28], release destructive catalytic enzymes, and damage the collagen matrix. Collagen content damage in the chorioamniotic sac leads to tearing, which in turn causes PROM and PPROM [29–31]. Similarly, exposure to tobacco smoke [32] or disinfection by-products in drinking water [33] has been linked to PROM and PPROM by oxidative stress.

The level of temperature variation with time, so the overlaps between temperature and season and PM2.5 may exist. Variance Inflation Factor test confirmed the multicollinearities between temperature and PM2.5 which may lead to overfitting. We also found no significant relationship between temperature and (P)PROM, therefore, in the main text we did not incorporate the temperature exposure into the adjustment model. Nevertheless, we conduct sensitivity analysis to temperature adjustment in the supplementary file. After adjustment for temperature, the results did not change considerably. The reason for the widen of the 95% CIs may be due to the inclusion of variables with severe multicollinearity, as well as the increased degree of model freedom due to the addition of adjustment variables. This is consistent with the sensitivity analysis of Pereira et al. [11] that temperature adjustment did not improve precision of the estimate between PM_2.5_ and PROM.

The present study has several strengths. First, the sample size was relatively large, which gave relatively good generalizability to the surrounding population. Second, we collected the past medical records of all births throughout the hospital to ensure unbiased choices. Third, given the different adjustments for mean PM_2.5_ in the other two trimesters in Model II, the result was notably disparate from that in Model I. As far as we know, this is the first study to adjust the independent variables in the other two trimesters. Our results can lead to more reliable research and may partly explain the negative results in previous studies. Fourth, we found the turning points of 46 and 63 μg/m^3^ for PROM and PPROM, respectively. This may not be accurate enough and require further confirmation in future studies for more sample sizes and different regions, but it can still provide a certain degree of reference for prenatal care, pollution control and further research. Fifth, previous studies can provide limited information for population in Asia or in areas with relatively high levels of air pollution, while our research partly filled this gap.

Several limitations should be noted in our study. First, some values were missing because data were based on fore-passed hospital records. Also, misclassification of (P)PROM may exist, since Nitrazine test for diagnosis of (P)PROM is a test with high sensitivity but poor specificity. But it is reasonable to consider the misclassification as independent and nondifferential, which would bias the observed association towards the null. Second, we used a single-ground-based PM_2.5_ monitor throughout the study period. Hence, the effect of exact street address and possible maternal residential mobility during pregnancy might have been overlooked. Nevertheless, the selection of a threshold distance of 40 km was reasonable, because PM_2.5_ can travel greater distances than PM_10_, whose exposure models are agree with estimates derived from the closest monitoring station [11, 34]. The same threshold distance was used in study of Pereira in 2014 [11]. Moreover, in a study of PM_2.5_ and mortality published in 2017, Di et al. found that estimates of risk based on ZIP-Code-specific assessments of exposure were slightly higher than those provided by the nearest data-monitoring site (within a distance of 50 km), these two measurement methods can get similar conclusions [13]. Therefore, it is plausible that such agreement would be better still for a threshold distance of 40 km in our study. Third, we used ambient pollutant levels as a surrogate for personal exposure, which might lead to some exposure biases. However, these should both result only in nondifferential exposure error and underestimates of pollutant mediated (P)PROM risks. Besides, we did not include some possible related factors (subclinical infections, etc.) in the analysis, however, the robustness of the sensitivity analysis results could give us more confidence in the associations we observed.

## Conclusion

In this cohort study, we found that for the first time exposure to PM_2.5_ during pregnancy was significantly associated with the risk of PROM and PPROM in Wuhan, Central China. These findings could provide further evidence for the adverse impact of air pollution on pregnancy outcomes and could also benefit public health to a certain degree. Further mechanism and intervention studies (such as antioxidant and so on) should be performed.

## Supplementary information

**Additional file 1:** **Fig S1~Fig S10.** Separate generalized additive models curves to estimate the relationships between the concentration of PM2.5 and the risk of PROM/PPROM. **Table S1.** Univariate analysis for association of the characteristics and the risk of (P)PROM. **Table S2.** Subgroup analysis. **Figs S11–S13** and **Tables S3-S7.** Sensitivity analysis about temperature.

## Data Availability

The datasets generated during the current study can be obtained from the corresponding authors upon reasonable request.

## Abbreviations

CI: Confidence interval
OR: Odds ratio
PM_2.5_: Airborne particulate matter with an aerodynamic diameter of 0.25 μm or less
PPROM: Preterm premature rupture of membranes
PROM: Premature rupture of membranes
