# Supplementary material for: Maternal exposure to ambient fine particulate matter and risk of premature rupture of membranes in Wuhan, Central China: a cohort study
Source: Environ Health. 2019 Nov 14;18:96. doi: 10.1186/s12940-019-0534-y (PMC6857323; doi:10.1186/s12940-019-0534-y)
Supplement: Supplementary file 1 — Additional file 1: Fig S1~Fig S10. Separate generalized additive models curves to estimate the relationships between the concentration of PM2.5 and the risk of PROM/PPROM. Table S1. Univariate analysis for association of the characteristics and the risk of (P)PROM. Table S2. Subgroup analysis. Figs S11–S13 and Tables S3-S7. Sensitivity analysis about temperature. [file 12940_2019_534_MOESM1_ESM.docx]

**Supplemental Material**

**Maternal Exposure to Ambient Fine Particulate Matter and Premature Rupture of Membranes in Chinese Population: A cohort study**

**Kun Wang^1^, Yu Tian^2^, Huabo Zheng****^1^, Shengshuai Shan^1^, XiaoFang Zhao^1^, Chengyun Liu^1,3*^**

We applied generalized additive models (GAM) to estimate the relationships between the concentration of PM_2.5_ for each trimester and the whole pregnancy and the risk of PROM/PPROM both without and with adjustment for confounding variables (Supplement Fig.S1~Fig. S6).

**
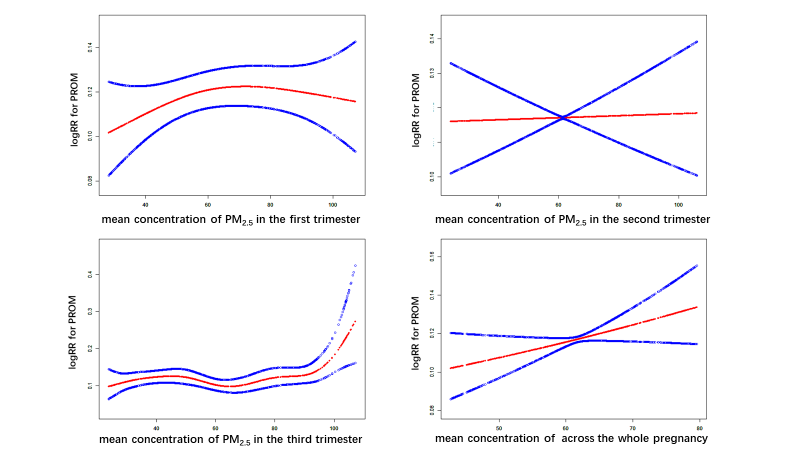
**

**Fig. S1 Unadjusted smooth curves between PM_2.5_ and PROM.**

The red lines indicate the estimated risk of incidence, and the blue lines represent point wise 95% confidence interval.


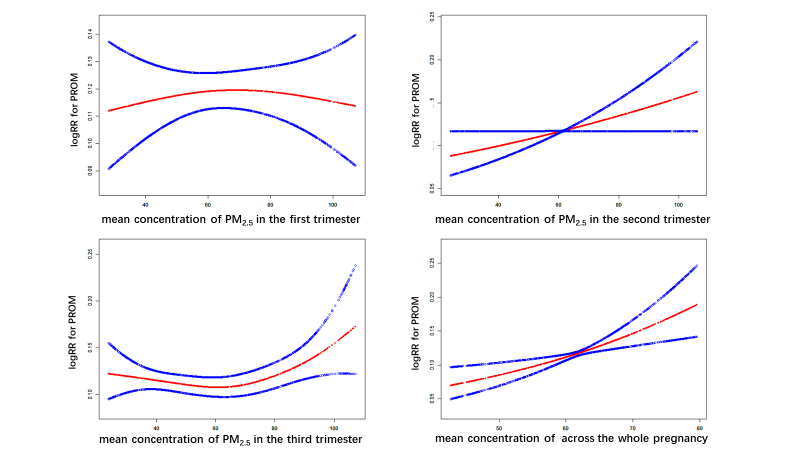


**Fig. S2 Smooth curves between PM_2.5_ and PROM after adjusting the confounding factors in Model Ⅰ*****.**

The red lines indicate the estimated risk of incidence, and the blue lines represent point wise 95% confidence interval.

^*^Model Ⅰ adjusted for: year of birth, season of conception, maternal age, parity, maternal anemia, preeclampsia, gestational diabetes, and history of obstetrical-gynecological pathology


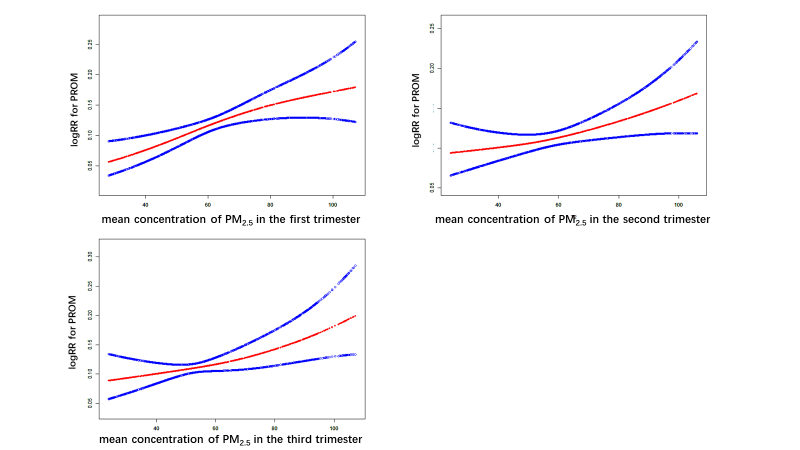


**Fig. S3 Smooth curves between PM_2.5_ and PROM after adjusting the confounding factors in Model Ⅱ******.**

The red lines indicate the estimated risk of incidence, and the blue lines represent point wise 95% confidence interval.

** Model Ⅱ adjusted for year of birth, season of conception, maternal age, parity, maternal anemia, preeclampsia, gestational diabetes, and history of obstetrical-gynecological pathology (Model Ⅰ) plus mean concentration of PM_2.5_ in the other two trimesters


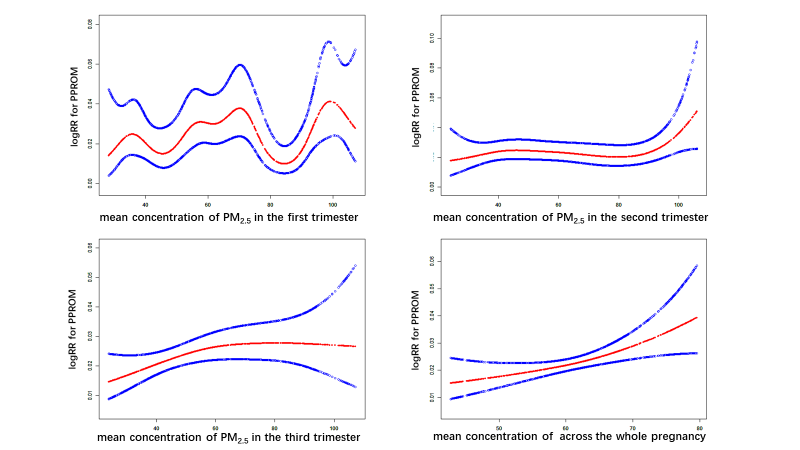


**Fig. S4 Unadjusted smooth curves between PM_2.5_ and PPROM.**

The red lines indicate the estimated risk of incidence, and the blue lines represent point wise 95% confidence interval.


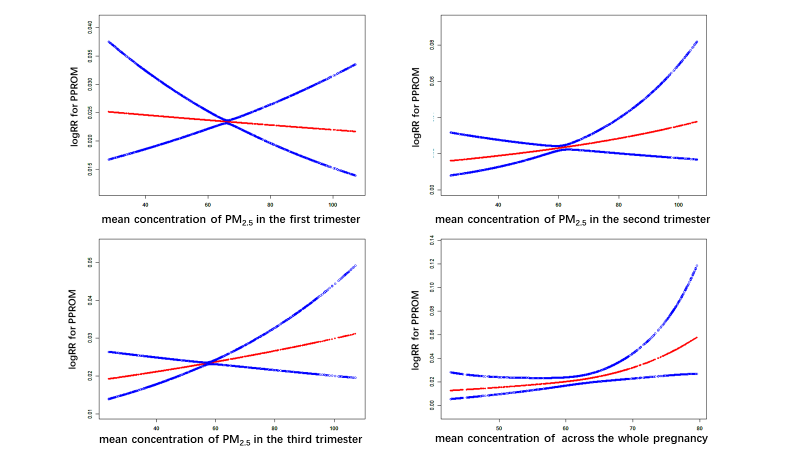


**Fig. S5 Smooth curves between PM_2.5_ and PPROM after adjusting the confounding factors in Model Ⅰ*****.**

The red lines indicate the estimated risk of incidence, and the blue lines represent point wise 95% confidence interval.

^*^Model Ⅰ adjusted for: year of birth, season of conception, maternal age, parity, maternal anemia, preeclampsia, gestational diabetes, and history of obstetrical-gynecological pathology


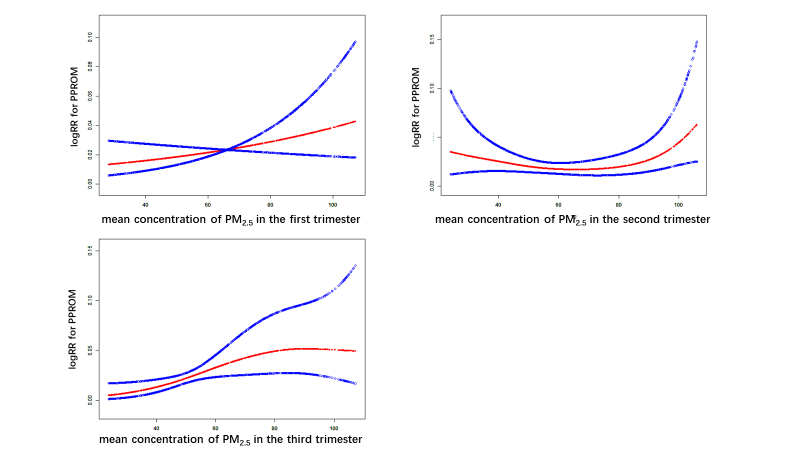


**Fig. S6 Smooth curves between PM_2.5_ and PPROM after adjusting the confounding factors in Model Ⅱ******.**

The red lines indicate the estimated risk of incidence, and the blue lines represent point wise 95% confidence interval.

** Model Ⅱ adjusted for year of birth, season of conception, maternal age, parity, maternal anemia, preeclampsia, gestational diabetes, and history of obstetrical-gynecological pathology (Model Ⅰ) plus mean concentration of PM_2.5_ in the other two trimesters

We applied generalized additive models (GAM) to estimate the relationships between the concentration of PM_2.5_ in the last week and day of pregnancy and the risk of PROM/PPROM both without and with adjustment for confounding variables (Supplement Fig.S7~Fig. S10).


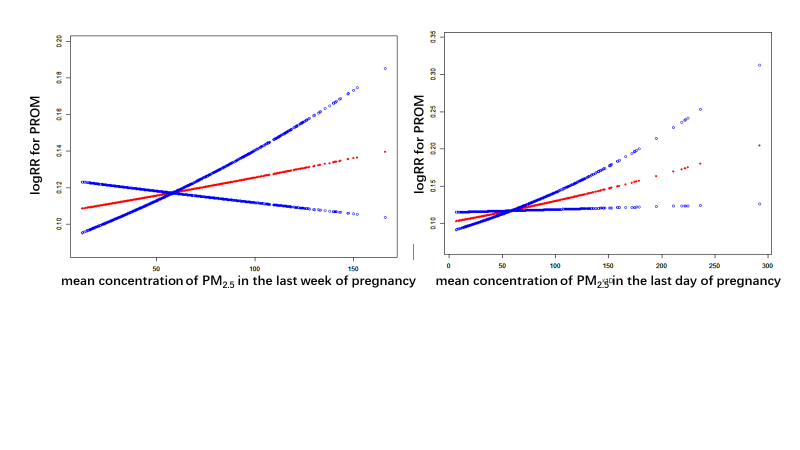


**Fig. S7 Unadjusted smooth curves between PM_2.5_ and PROM.**

The red lines indicate the estimated risk of incidence, and the blue lines represent point wise 95% confidence interval.


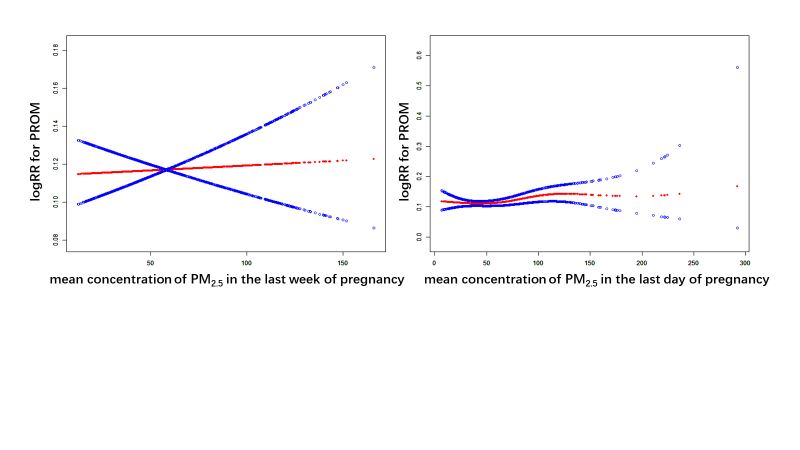


**Fig. S8 Adjusted smooth curves between PM_2.5_ and PROM*****.**

The red lines indicate the estimated risk of incidence, and the blue lines represent point wise 95% confidence interval.

*Adjusted for: year of birth, season of conception, maternal age, parity, maternal anemia, preeclampsia, gestational diabetes, and history of obstetrical-gynecological pathology.


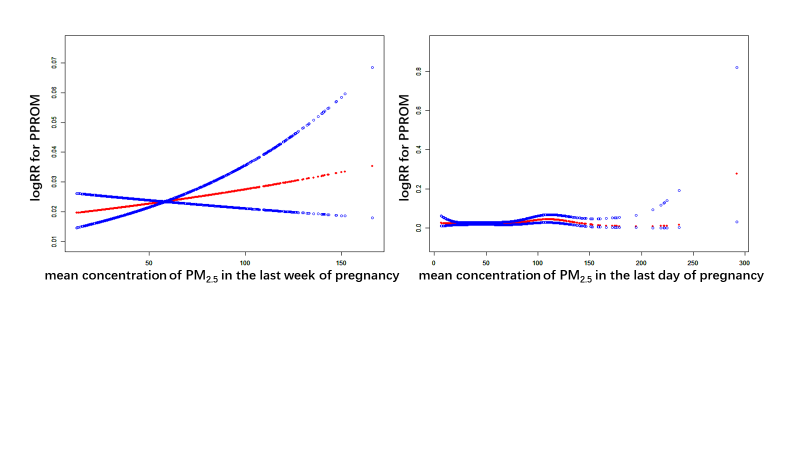


**Fig. S9 Unadjusted smooth curves between PM_2.5_ and PPROM.**

The red lines indicate the estimated risk of incidence, and the blue lines represent point wise 95% confidence interval.


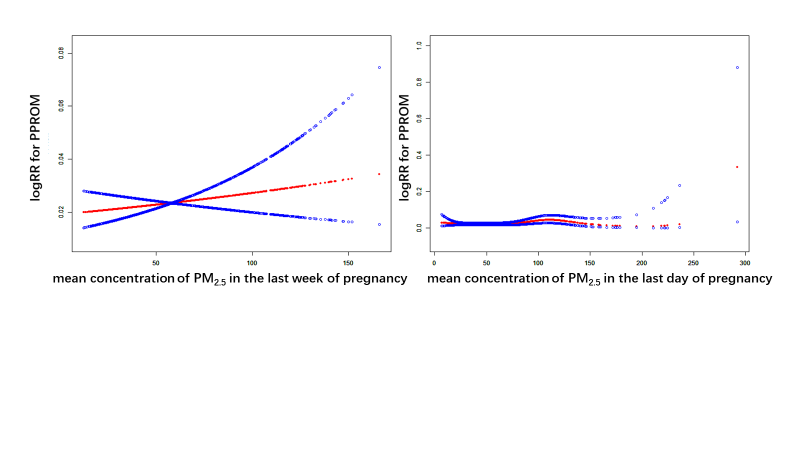


**Fig. S10 Adjusted smooth curves between PM_2.5_ and PPROM*****.**

The red lines indicate the estimated risk of incidence, and the blue lines represent point wise 95% confidence interval.

* Adjusted for: year of birth, season of conception, maternal age, parity, maternal anemia, preeclampsia, gestational diabetes, and history of obstetrical-gynecological pathology.

**Table S1.** **Univariate analysis for association of the characteristics and the risk of (P)PROM.**

| Characteristics | PROM  (*N*=511) | | PPROM  (*N*=102) | |
| --- | --- | --- | --- | --- |
|  | OR (95% CI) | *P* value | OR (96%CI) | *P* value |
| Mode of delivery |  |  |  |  |
| Vaginal | 1.0 |  | 1.0 |  |
| Cesarean | 0.84 (0.67, 1.05) | 0.1203 | 0.48 (0.27, 0.85) | 0.0119 |
| Year of birth |  |  |  |  |
| 2015 | 1.0 |  | 1.0 |  |
| 2016 | 0.93 (0.75, 1.15) | 0.5004 | 0.91 (0.58, 1.40) | 0.6552 |
| 2017 | 0.76 (0.60, 0.97) | 0.0291 | 0.43 (0.23, 0.79) | 0.0063 |
| Season of conception^a^ |  |  |  |  |
| Spring | 1.0 |  | 1.0 |  |
| Summer | 0.97 (0.73, 1.27) | 0.8095 | 0.99 (0.54, 1.81) | 0.9736 |
| Autumn | 0.88 (0.67, 1.15) | 0.3442 | 1.01 (0.57, 1.81) | 0.9687 |
| Winter | 1.14 (0.88, 1.48) | 0.3312 | 1.09 (0.61, 1.93) | 0.7737 |
| Maternal age | 0.99 (0.97, 1.02) | 0.5725 | 0.98 (0.93, 1.04) | 0.4667 |
| Maternal age group |  |  |  |  |
| 18-19 | 1.0 |  | 1.0 |  |
| 20-24 | 1.22 (0.51, 2.87) | 0.6560 | 0.41 (0.12, 1.42) | 0.1602 |
| 25-29 | 1.39 (0.60, 3.27) | 0.4437 | 0.53 (0.16, 1.75) | 0.2981 |
| 30-35 | 1.08 (0.46, 2.58) | 0.8547 | 0.35 (0.10, 1.23) | 0.1002 |
| Parity |  |  |  |  |
| 0 | 1.0 |  | 1.0 |  |
| ≥1 | 0.54 (0.39, 0.75) | 0.0002 | 0.39 (0.17, 0.90) | 0.0263 |
| Maternal anemia |  |  |  |  |
| No | 1.0 |  | 1.0 |  |
| Yes | 0.99 (0.69, 1.41) | 0.9468 | 0.97 (0.44, 2.11) | 0.9356 |
| Maternal preeclampsia |  |  |  |  |
| No | 1.0 |  | 1.0 |  |
| Yes | 1.47 (0.72, 3.03) | 0.2925 | 0.79 (0.11, 5.80) | 0.8201 |
| Maternal gestational diabetes |  |  |  |  |
| No | 1.0 |  | 1.0 |  |
| Yes | 0.95 (0.60, 1.51) | 0.8296 | 3.24 (1.74, 6.05) | 0.0002 |
| History of obstetrical-gynecological pathology^b^ |  |  |  |  |
| No | 1.0 |  | 1.0 |  |
| Yes | 0.78 (0.47, 1.27) | 0.3127 | 0.45 (0.11, 1.82) | 0.2609 |
| Mean temperature across the whole pregnancy (°C) | 1.0 (1.0, 1.0) | 0.960 | 1.0 (0.9, 1.1) | 0.947 |
| Mean concentration of PM2.5 in the 1st trimester (µg/m^3^) | 1.0 (1.0, 1.1) | 0.400 | 1.0 (0.9, 1.1) | 0.680 |
| Mean concentration of PM2.5 in the 2nd trimester (µg/m^3^) | 1.0 (1.0, 1.0) | 0.866 | 1.0 (1.0, 1.1) | 0.325 |
| Mean concentration of PM2.5 in the 3rd trimester (µg/m^3^) | 1.0 (1.0, 1.1) | 0.148 | 1.1 (1.0, 1.2) | 0.134 |
| Mean concentration of PM2.5 across the whole pregnancy (μg/m^3^) | 1.1 (1.0, 1.2) | 0.067 | 1.3 (1.0, 1.6) | 0.029 |

OR: Odds Ratio; CI: Confidence Interval

PROM: Premature rupture of membranes; PPROM: Preterm premature rupture of membranes

^a^Spring: March to May; Summer: June to August; Autumn: September to November; Winter: December to February

^b^Including chorioamnionitis, uterine myoma, adnexal cyst, pelvic infection, pelvic pathologic adhesion, pelvicellulitis, cervicitis, vaginal bleeding during pregnancy, and/or colpomycosis.

**Sensitivity analysis 1：subgroup analysis**

**Table S2. ORs (95%CI) for PROM and PPROM per 10 μg/m^3^ Increase in PM2.5 Across the Whole Pregnancy** **in subgroups of age, Parity, mode of delivery, season of conception, maternal anemia, preeclampsia, gestational diabetes and history of obstetrical-gynecological pathology.** ^*^ **(*N*=4364)**

|  |  | PROM  (*N*=511) | | | PPROM  (*N*=102) | | |  |
| --- | --- | --- | --- | --- | --- | --- | --- | --- |
|  | total No. | No. of  events | OR (95% CI) | *P* value | No. of  events | OR (95% CI) | *P* value | |
| Maternal age group |  |  |  |  |  |  |  | |
| 18-24 | 1278 | 142 | 1.57 (1.11, 2.22) | 0.0103 | 29 | 2.88 (1.34, 6.22) | 0.0070 | |
| 25-29 | 2129 | 272 | 1.25 (0.97, 1.62) | 0.0887 | 56 | 1.09 (0.63, 1.87) | 0.7612 | |
| 30-35 | 957 | 97 | 1.32 (0.84, 2.07) | 0.2251 | 17 | 1.20 (0.47, 3.07) | 0.7075 | |
| Parity |  |  |  |  |  |  |  | |
| 0 | 3751 | 467 | 1.33 (1.09, 1.61) | 0.0045 | 96 | 1.62 (1.07, 2.45) | 0.0223 | |
| ≥1 | 613 | 44 | 1.75 (0.89, 3.47) | 0.1072 | 6 | 0.90 (0.16, 5.15) | 0.9025 | |
| Mode of delivery |  |  |  |  |  |  |  | |
| Vaginal | 3267 | 396 | 1.32 (1.07, 1.63) | 0.0085 | 88 | 1.53 (1.01, 2.33) | 0.0441 | |
| Cesarean | 1097 | 115 | 1.49 (0.96, 2.30) | 0.0723 | 14 | 2.09 (0.53, 8.34) | 0.2946 | |
| Season of conception^a^ |  |  |  |  |  |  |  | |
| Spring/Winter | 2205 | 274 | 1.28 (1.05, 1.57) | 0.0165 | 54 | 1.36 (0.89, 2.08) | 0.1569 | |
| Summer/Autumn | 2159 | 237 | 1.91 (1.12, 3.24) | 0.0167 | 48 | 2.42 (0.74, 7.84) | 0.1418 | |
| Maternal anemia |  |  |  |  |  |  |  | |
| No | 4045 | 474 | 1.35 (1.11, 1.63) | 0.0021 | 95 | 1.46 (0.97, 2.18) | 0.0693 | |
| Yes | 319 | 37 | 1.21 (0.47, 3.11) | 0.6931 | 7 | 5.48 (0.41, 72.4) | 0.1967 | |
| Maternal preeclampsia |  |  |  |  |  |  |  | |
| No | 4308 | 490 | 1.37 (1.14, 1.65) | 0.0010 | 90 | 1.52 (1.03, 2.26) | 0.0359 | |
| Yes | 56 | 21 | -^ | -^ | 12 | -^ | -^ | |
| Maternal gestational diabetes |  |  |  |  |  |  |  | |
| No | 4175 | 502 | 1.36 (1.12, 1.64) | 0.0017 | 101 | 1.55 (1.01, 2.36) | 0.0428 | |
| Yes | 189 | 9 | 1.05 (0.35, 3.19) | 0.9306 | 1 | 1.78 (0.34, 9.37) | 0.4957 | |
| History of obstetrical-gynecological pathology^b^ |  |  |  |  |  |  |  | |
| No | 4173 | 493 | 1.32 (1.10, 1.60) | 0.0036 | 100 | 1.51 (1.02, 2.25) | 0.0398 | |
| Yes | 191 | 18 | 5.42 (0.98, 29.9) | 0.0526 | 2 | -^ | -^ | |

PROM: Premature rupture of membranes; PPROM: Preterm premature rupture of membranes； OR: Odds Ratio; CI: Confidence Interval

^*^Adjusted for: year of birth, season of conception, maternal age, parity, maternal anemia, preeclampsia, gestational diabetes, and history of obstetrical-gynecological pathology

^a^Spring: March to May; Summer: June to August; Autumn: September to November; Winter: December to February

^b^Including chorioamnionitis, uterine myoma, adnexal cyst, pelvic infection, pelvic pathologic adhesion, pelvicellulitis, cervicitis, vaginal bleeding during pregnancy, and/or colpomycosis.

^The model failed because of the small sample size.

**Sensitivity analysis 2：About** **temperature**


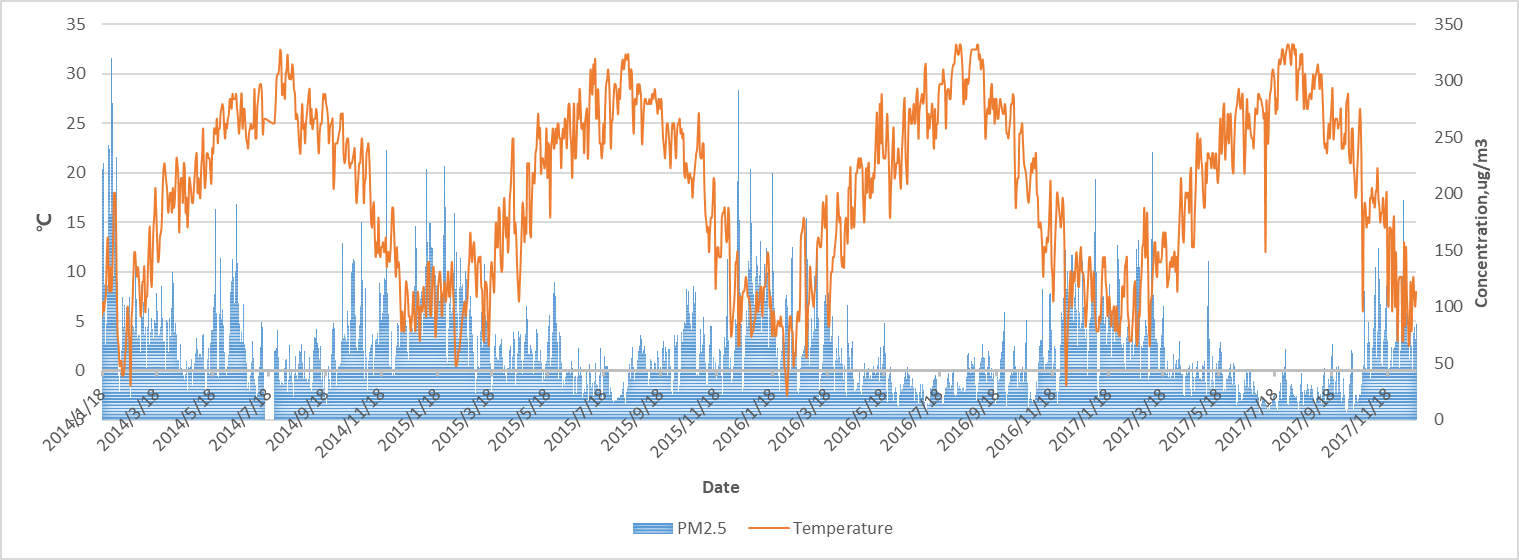


**Fig. S11 Temporal trend of PM2.5 and temperature level from January 2014 to December 2017.**


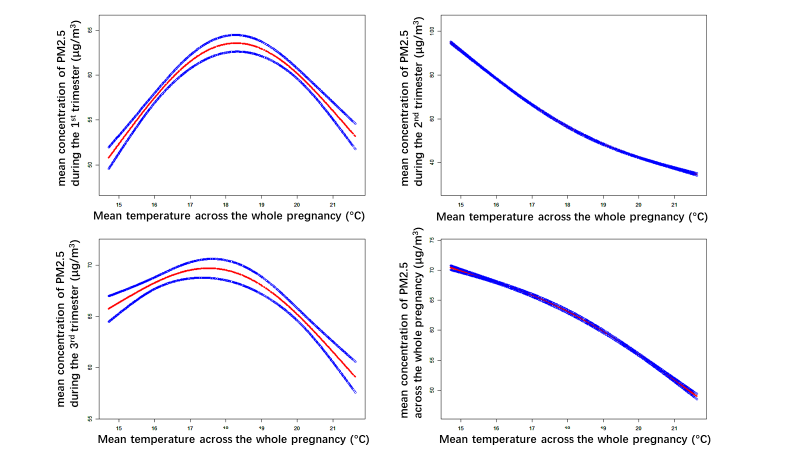


**Fig. S12 Unadjusted smooth curves between temperature and PM_2.5_.**

The red lines indicate the estimated risk of incidence, and the blue lines represent point wise 95% confidence interval.


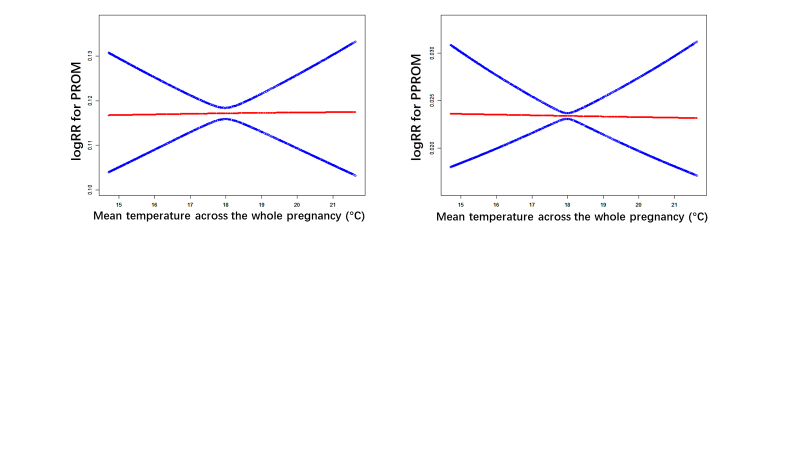


**Fig. S13 Unadjusted smooth curves between temperature and (P)PROM.**

The red lines indicate the estimated risk of incidence, and the blue lines represent point wise 95% confidence interval.

**Table S3. ORs (95% CI) for PROM and PPROM associated with 1℃ Increase in Mean Temperature Across the Whole Pregnancy (*N*=4364)**

|  | Crude | Model Ⅰ^*^ |
| --- | --- | --- |
| PROM |  |  |
| Last week of pregnancy | 1.00 (0.96, 1.04) | 1.01 (0.97, 1.05) |
| PPROM |  |  |
| Last week of pregnancy | 1.00 (0.92, 1.08) | 0.98 (0.90, 1.07) |

OR: Odds Ratio; CI: Confidence Interval

PROM: Premature rupture of membranes; PPROM: Preterm premature rupture of membranes

Adjusted for: year of birth, maternal age, parity, maternal anemia, preeclampsia, gestational diabetes, history of obstetrical-gynecological pathology and mean temperature across the whole pregnancy.

**Table S4.** **Variance Inflation Factor Test for Multicollinearity.**

|  | 1st trimester | 2nd trimester | 3rd trimester | Whole pregnancy |
| --- | --- | --- | --- | --- |
| Year of birth | 1.4 | 1.6 | 1.1 | 3.6 |
| Season of conception | 5.3 | 5.1 | 5.2 | 5.2 |
| Maternal age | 1.1 | 1.1 | 1.1 | 1.1 |
| Parity | 1.2 | 1.2 | 1.2 | 1.2 |
| Maternal anemia | 1 | 1 | E | 1 |
| Preeclampsia | 1 | 1 | 1 | 1 |
| Gestational diabetes | 1 | 1 | 1 | 1 |
| History of obstetrical-gynecological pathology | 1.1 | 1.1 | 1.1 | 1.1 |
| Mean temperature across the whole pregnancy (°C) | 5.3 | 16.1 | 5.2 | 10.8 |

**Table S5. ORs (95%CI) for PROM and PPROM per 10 μg/m^3^ Increase in PM2.5 in Each Trimester and Across the Whole Pregnancy (*N*=4364)**

|  | Crude | Model Ⅰ^*^ | Model Ⅱ^**^ |
| --- | --- | --- | --- |
| PROM |  |  |  |
| 1st trimester | 1.02 (0.97, 1.06) | 0.99 (0.94, 1.04) | 1.13 (0.98, 1.30) ^†^ |
| 2nd trimester | 1.00 (0.96, 1.05) | 1.00 (0.85, 1.17) | 1.07 (0.84, 1.35) ^‡^ |
| 3rd trimester | 1.03 (0.99, 1.08) | 1.03 (0.99, 1.08) | 1.13 (1.02, 1.25) ^§^ |
| Whole pregnancy | 1.09 (0.99, 1.20) | 1.39 (1.05, 1.84) |  |
| PPROM |  |  |  |
| 1st trimester | 1.04 (0.95, 1.13) | 0.96 (0.86, 1.08) | 1.28 (0.96, 1.71) ^†^ |
| 2nd trimester | 1.05 (0.96, 1.14) | 1.16 (0.82, 1.65) | 1.39 (0.83, 2.33) ^‡^ |
| 3rd trimester | 1.07 (0.98, 1.18) | 1.08 (0.98, 1.18) | 1.24 (1.02, 1.52) ^§^ |
| Whole pregnancy | 1.31 (1.06, 1.62) | 1.90 (1.09, 3.30) |  |

OR: Odds Ratio; CI: Confidence Interval

PROM: Premature rupture of membranes; PPROM: Preterm premature rupture of membranes

^*^Model Ⅰ adjusted for: year of birth, season of conception, maternal age, parity, maternal anemia, preeclampsia, gestational diabetes, history of obstetrical gynecological pathology and mean temperature across the whole pregnancy.

^**^Model Ⅱ adjusted for Model Ⅰ plus mean concentration of PM2.5 in the other two trimesters

^†^ Model Ⅰ plus mean concentration of PM2.5 in the 2nd and 3rd trimesters

^‡^ Model Ⅰ plus mean concentration of PM2.5 in the 1st and 3rd trimesters

^§^ Model Ⅰ plus mean concentration of PM2.5 in the 1st and 2nd trimesters

**Table S6. Threshold Effect for PROM and PPROM per 10 μg/m^3^ Increase in PM2.5 Across the Whole Pregnancy. (N=4364)** ^*^

|  | ORs (95%CI) |
| --- | --- |
| PROM |  |
| PM2.5 < 46 (μg/m^3^) | 0.19 (0.03, 1.39) |
| PM2.5 ≥ 46 (μg/m^3^) | 1.46 (1.10, 1.95) |
| PPROM |  |
| PM2.5 < 63 (μg/m^3^) | 1.38 (0.72, 2.63) |
| PM2.5 ≥ 63 (μg/m^3^) | 2.79 (1.40, 5.58) |

OR: Odds Ratio; CI: Confidence Interval

PROM: Premature rupture of membranes; PPROM: Preterm premature rupture of membranes

^*^Adjusted for: year of birth, season of conception, maternal age, parity, maternal anemia, preeclampsia, gestational diabetes, history of obstetrical-gynecological pathology and mean temperature across the whole pregnancy. (Model Ⅰ)

**Table S7. ORs (95% CI) for PROM and PPROM associated with 10 µg/m^3^ Increase in PM2.5 in the last week and last day of pregnancy (*N*=4364)** ^*^

|  | Crude | Model Ⅰ^*^ |
| --- | --- | --- |
| PROM |  |  |
| Last day of pregnancy | 1.03 (1.00, 1.05) | 1.03 (1.00, 1.06) |
| Last week of pregnancy | 1.02 (0.99, 1.05) | 1.02 (0.98, 1.06) |
| PPROM |  |  |
| Last day of pregnancy | 1.04 (0.99, 1.10) | 1.06 (1.00, 1.12) |
| Last week of pregnancy | 1.04 (0.98, 1.11) | 1.06 (0.97, 1.14) |

OR: Odds Ratio; CI: Confidence Interval

PROM: Premature rupture of membranes; PPROM: Preterm premature rupture of membranes

^*^Adjusted for: year of birth, season of conception, maternal age, parity, maternal anemia, preeclampsia, gestational diabetes, history of obstetrical-gynecological pathology and mean temperature across the whole pregnancy. (Model Ⅰ)
